# Supplementary material for: Ultra-Processed Food Consumption and Its Association with Risk of Obesity, Sedentary Behaviors, and Well-Being in Adolescents
Source: Nutrients. 2024 Nov 8;16(22):3827. doi: 10.3390/nu16223827 (PMC11597557; doi:10.3390/nu16223827)
Supplement: Supplementary file 1 [file nutrients-16-03827-s001.zip › nutrients-3298784-supplementary.pdf]

SUPPLEMENTARY MATERIALS

Table S1. Mean difference by sex, standard error and 95% bootstrap confidence intervals (CI) for the main analyzed variables

Variables	Mean difference	Standard error	95% bootstrap CI *
**NOVA-UPF 24h recall**			
Drinks and yogurts	0.34	0.22	−0.09 to 0.76
Packaged and fast food	0.33	0.30	−0.24 to 0.89
Sweet and salty snacks	−0.14	0.26	−0.64 to 0.36
Total score	0.54	0.61	−0.64 to 1.69
**NOVA-UPF away from home**			
Drinks and yogurts	0.36	0.22	−0.08 to 0.80
Packaged and fast food	0.42	0.33	−0.25 to 1.08
Sweet and salty snacks	0.31	0.28	−0.23 to 0.85
Total score	1.09	0.76	−0.40 to 2.54
Body mass index (kg/m^2)	−1.91	0.54	−3.01 to −0.83
Body fat (%)	−8.30	1.00	−10.26 to −6.31
**Sedentary time during weekdays**			
Total time. min/day	−0.35	0.33	−1.00 to 0.29
Watching TV. min/day	0.01	10.24	−19.26 to 20.36
Using a PC. min/day	10.89	10.18	−8.34 to −31.02
Playing electronic games. min/day	45.60	15.26	19.80 to 76.99
Using a smartphone. min/day	−39.70	25.99	−90.67 to 12.41
Using tablets. min/day	−2.01	7.00	−14.87 to 11.53
Studying. min/day	−31.75	11.59	−55.02 to −8.02
Other activities. min/day	−14.08	9.66	−37.13 to 3.72
**Sedentary time at the weekend**			
Total time. min/day	−0.11	0.48	−1.02 to 0.86
Watching TV. min/day	2.25	16.46	−29.06 to 34.26
Using a PC. min/day	42.75	18.48	6.08 to 79.56
Playing electronic games. min/day	88.55	22.14	50.28 to 133.18
Using a smartphone. min/day	−27.58	35.43	−92.09 to 44.41
Using tablets. min/day	1.12	8.70	−14.63 to 17.80
Studying. min/day	−31.81	11.48	−53.78 to −9.52
Other activities. min/day	−15.68	7.29	−31.24 to −2.12
**Emotional well-being**	1.28	0.36	0.60 to 2.00
**Social well-being**	2.71	0.71	1.30 to 4.13
**Psychological well-being**	2.67	0.76	1.13 to 4.20
**Physical well-being**	2.96	0.42	2.17 to 3.76

Note: 95% bias-corrected and accelerated bootstrap (BCa) confidence intervals were estimated from 5000 bootstrap samples (seed 151024); * Differences were considered significant if the 95% bootstrap CI of the mean difference (males' score minus females' score) did not include zero.

Table S2. 95% bootstrap confidence intervals (CI) of Pearson correlations between the main study variables

Variables	NOVA-UPF 24h recall *				NOVA-UPF away from home *			
	DY (95% CI *r*)	PFF (95% CI *r*)	SSS (95% CI *r*)	Total (95% CI *r*)	DY (95% CI *r*)	PFF (95% CI *r*)	SSS (95% CI *r*)	Total (95% CI *r*)
Body mass index	−0.17 to 0.05	−0.16 to 0.03	−0.18 to 0.03	−0.19 to 0.01	−0.19 to 0.05	−0.21 to −0.04	−0.19 to −0.01	−0.21 to −0.02
Body fat	−0.24 to −0.01	−0.22 to 0.03	−0.21 to 0.01	−0.25 to −0.02	−0.24 to −0.03	−0.21 to −0.02	−0.23 to −0.04	−0.23 to −0.05
**Sedentary time during weekdays**								
Total time	−0.03 to 0.21	−0.01 to 0.23	−0.08 to 0.15	−0.02 to 0.22	−0.03 to 0.24	−0.04 to 0.23	−0.01 to 0.23	−0.02 to 0.25
Watching TV	−0.04 to 0.25	−0.06 to 0.36	−0.12 to 0.17	−0.04 to 0.29	0.00 to 0.22	−0.03 to 0.24	−0.03 to 0.21	−0.01 to 0.24
Using a PC	−0.12 to 0.13	−0.14 to 0.10	−0.06 to 0.17	−0.11 to 0.13	−0.02 to 0.21	−0.06 to 0.16	−0.05 to 0.19	−0.03 to 0.19
Playing electronic games	0.02 to 0.32	−0.01 to 0.41	−0.08 to 0.27	0.03 to 0.34	−0.07 to 0.18	−0.11 to 0.04	−0.10 to 0.11	−0.09 to 0.09
Using a smartphone	0.08 to 0.32	0.08 to 0.40	−0.03 to 0.26	0.11 to 0.35	0.01 to 0.22	−0.06 to 0.25	−0.03 to 0.22	−0.02 to 0.25
Using tablets	−0.09 to 0.12	−0.11 to 0.11	−0.03 to 0.21	−0.05 to 0.13	−0.13 to 0.04	−0.10 to 0.10	−0.06 to 0.10	−0.09 to 0.08
Studying	−0.24 to −0.02	−0.26 to −0.09	−0.11 to 0.11	−0.23 to −0.03	−0.20 to 0.05	−0.14 to 0.10	−0.15 to 0.03	−0.16 to 0.06
Other activities	−0.15 to 0.03	−0.11 to 0.07	−0.13 to 0.06	−0.13 to 0.04	−0.09 to 0.15	−0.10 to 0.08	−0.08 to 0.14	−0.10 to 0.13
**Sedentary time during weekend days**								
Total time	−0.03 to 0.22	0.00 to 0.24	−0.09 to 0.16	−0.01 to 0.23	0.01 to 0.25	−0.09 to 0.12	−0.03 to 0.19	−0.03 to 0.19
Watching TV	−0.04 to 0.26	0.08 to 0.45	−0.14 to 0.15	0.02 to 0.32	0.00 to 0.20	−0.04 to 0.24	−0.06 to 0.21	−0.03 to 0.23
Using a PC	−0.19 to 0.02	−0.13 to 0.07	−0.09 to 0.11	−0.15 to 0.06	−0.07 to 0.16	−0.11 to 0.10	−0.08 to 0.13	−0.09 to 0.12
Playing electronic games	0.06 to 0.34	0.06 to 0.44	−0.12 to 0.17	0.05 to 0.34	−0.06 to 0.13	−0.10 to 0.12	−0.13 to 0.05	−0.09 to 0.09
Using a smartphone	−0.07 to 0.22	−0.01 to 0.31	−0.06 to 0.17	−0.03 to 0.26	−0.10 to 0.14	−0.08 to 0.16	−0.05 to 0.17	−0.07 to 0.16
Using tablets	−0.11 to 0.10	−0.11 to 0.10	−0.04 to 0.21	−0.06 to 0.11	−0.10 to 0.06	−0.08 to 0.14	−0.07 to 0.11	−0.09 to 0.12
Studying	−0.30 to −0.07	−0.27 to −0.07	−0.13 to 0.10	−0.26 to −0.05	−0.24 to −0.01	−0.17 to 0.05	−0.16 to 0.04	−0.19 to 0.02
Other activities	−0.19 to 0.04	−0.07 to 0.19	−0.15 to 0.14	−0.15 to 0.15	−0.10 to 0.14	−0.11 to 0.10	−0.09 to 0.12	−0.10 to 0.12
Emotional well-being	−0.11 to 0.15	−0.12 to 0.10	−0.16 to 0.12	−0.13 to 0.12	−0.15 to 0.08	−0.12 to 0.13	−0.16 to 0.12	−0.14 to 0.11
Social well-being	0.00 to 0.23	−0.07 to 0.15	−0.10 to 0.16	−0.04 to 0.19	−0.05 to 0.19	0.00 to 0.24	−0.03 to 0.21	−0.02 to 0.23
Psychological well-being	−0.04 to 0.20	−0.06 to 0.17	−0.09 to 0.15	−0.05 to 0.19	−0.08 to 0.17	−0.03 to 0.22	−0.07 to 0.20	−0.05 to 0.21
Physical well-being	−0.10 to 0.15	−0.17 to 0.09	−0.23 to 0.04	−0.18 to 0.07	−0.09 to 0.16	−0.07 to 0.18	−0.15 to 0.12	−0.10 to 0.16

Note: DY= Drinks and yogurts; PFF= Packaged and fast food; SSS= Sweet and salty snacks; Total = Total UPF NOVA Score; 95% bias-corrected and accelerated bootstrap (BCa) confidence intervals were estimated from 5000 bootstrap samples (seed 151024); * Correlations were considered significant if the 95% bootstrap CI of the Pearson correlation did not include zero.
